# POSITION OF THE SBCBM - NOMENCLATURE AND DEFINITION OF OUTCOMES OF BARIATRIC AND METABOLIC SURGERY

**DOI:** 10.1590/S0102-6720201500S100002

**Published:** 2015-12

**Authors:** Luis V BERTI, Josemberg CAMPOS, Almino RAMOS, Marçal ROSSI, Thomas SZEGO, Ricardo COHEN

**Affiliations:** Brazilian Society of Bariatric and Metabolic Surgery

Obesity was initially regarded as a psychosocial disorder, but, after years of study, has come to be understood as a chronic disease for which there is no cure, like hypertension and diabetes^6^^,^ 7. Surgical intervention is safe and effective in the long term and aims to control the disease and its comorbidities. Even so, it is hoped that the proportion of patients who experience long-term recidivism can be reduced^1^^,^ 3. 

In the past 20 years, surgical treatment of obesity has evolved significantly and is regarded as more than an isolated modification of the digestive tract. This operation, in combination with changes in lifestyle, is fundamental for controlling the disease in the long and medium term. These changes can be achieved with appropriate guidance and follow-up from a multi-professional team^5^. 

 Comorbidities, such as diabetes, arterial hypertension, sleep apnea and others, reduce quality of life and increase mortality. Weight loss and control of these disease save lives and bring down the cost to the healthcare system^2,^ 4 ^,^ 8.

There is no consensus in the literature as to the definition of successful bariatric and metabolic surgery. The same outcome can be considered favorable by one author and unfavorable by another. There is also no unanimity as which treatment option is most appropriate for turning round a failed bariatric surgery. 

With a view to drawing up norms as to what constitutes successful bariatric surgery, the Brazilian Society of Bariatric and Metabolic Surgery-SBCBM-held a discussion forum involving surgeons, endocrinologists, cardiologists, nutritionists, psychiatrists, and physical exercise professionals. After critical analysis of the available literature in the light of the prior experience of participants, objective classification criteria were drawn up.

The following is a list of the final decisions reached by the panel of experts:

1. A patient who does not manage adequately to control obesity is different from one who experiences a relapse after various years of adequate control.

2. A small long-term weight gain is normal and to be expected after bariatric and metabolic surgery. 

3. The control of metabolic diseases and the consequent improvement in quality of life are the desired outcomes.

4. It is appropriate for the criteria for successful or unsuccessful surgery to employ the following terms:

- **Controlled obesity**: patients who achieve a Total Weight Loss of > 20% in 6 months;
- **Partially controlled obesity**: Total Weight Loss of between 10 and 20% in 6 months;
- **Uncontrolled obesity**: Total Weight Loss of < 10% in 6 months.

5. Along with the definition of Obesity Control, the following factors should also be taken into consideration:

- Patient satisfaction with the outcome;
- Improvement of associated diseases, irrespective of weight loss;
- Any weight also occurring prior to surgery.

6. For those patients who gain weight after a long period of control or those in whom an associated disease has reappeared the correct term is obesity recidivism, classified as follows:

- **Recidivism:** 50% of weight lost regained in long term or 20% of weight regained in association with reappearance of comorbidities.
- **Controlled Recidivism:** Between 20 and 50% of weight loss regained in long term.

NB. Expected weight gain: < 20% of weight loss regained in long term.

7. The causes of post-operative recidivism are related to factors relating to the patient (behavioral and biological) and surgical techniques.

8. The causes related to recidivism, especially behavioral ones, should first be evaluated by a multidisciplinary team and then the technical causes should be solved surgically.

9. We consider the ideal multidisciplinary team to comprise:

- an endocrinologist
- a surgeon
- a nutritional doctor
- a psychiatrist
- a nutritionist
- a psychologist
- a physical trainer
- a physiotherapist
- and other professionals if necessary.

Always leading the way, the SBCBM has established guidelines to help orient the outcomes of bariatric and metabolic surgery. Standardized terms and criteria will lead to standardized practices and provide better treatment for our patients. 

It is important to note that this document should not be used for legal purposes, as it does not deal with legislation on the subject, aiming only to provide instrutions for members of the society. 
